# The Neuronal Transition Probability (NTP) Model for the Dynamic Progression of Non-REM Sleep EEG: The Role of the Suprachiasmatic Nucleus

**DOI:** 10.1371/journal.pone.0023593

**Published:** 2011-08-19

**Authors:** Helli Merica, Ronald D. Fortune

**Affiliations:** 1 Laboratoire de Sommeil et de Neurophysiologie, Hôpitaux Universitaires de Genève, Belle Idée, Geneva, Switzerland; 2 CERN European Organisation for Nuclear Research, Geneva, Switzerland; Vanderbilt University, United States of America

## Abstract

Little attention has gone into linking to its neuronal substrates the dynamic structure of non-rapid-eye-movement (NREM) sleep, defined as the pattern of time-course power in all frequency bands across an entire episode. Using the spectral power time-courses in the sleep electroencephalogram (EEG), we showed in the typical first episode, several moves *towards*-and-*away* from deep sleep, each having an identical pattern linking the major frequency bands beta, sigma and delta. The neuronal transition probability model (NTP) – in fitting the data well – successfully explained the pattern as resulting from stochastic transitions of the firing-rates of the thalamically-projecting brainstem-activating neurons, alternating between two steady dynamic-states (*towards*-and-*away* from deep sleep) each initiated by a so-far unidentified flip-flop. The aims here are to identify this flip-flop and to demonstrate that the model fits well all NREM episodes, not just the first. Using published data on suprachiasmatic nucleus (SCN) activity we show that the SCN has the information required to provide a threshold-triggered flip-flop for **timing** the *towards*-and-*away* alternations, information provided by sleep-relevant feedback to the SCN. NTP then determines the **pattern** of spectral power within each dynamic-state. NTP was fitted to individual NREM episodes 1–4, using data from 30 healthy subjects aged 20–30 years, and the quality of fit for each NREM measured. We show that the model fits well all NREM episodes and the best-fit probability-set is found to be effectively the same in fitting all subject data. The significant model-data agreement, the constant probability parameter and the proposed role of the SCN add considerable strength to the model. With it we link for the first time findings at cellular level and detailed time-course data at EEG level, to give a coherent picture of NREM dynamics over the entire night and over hierarchic brain levels all the way from the SCN to the EEG.

## Introduction

**Figure 1:**
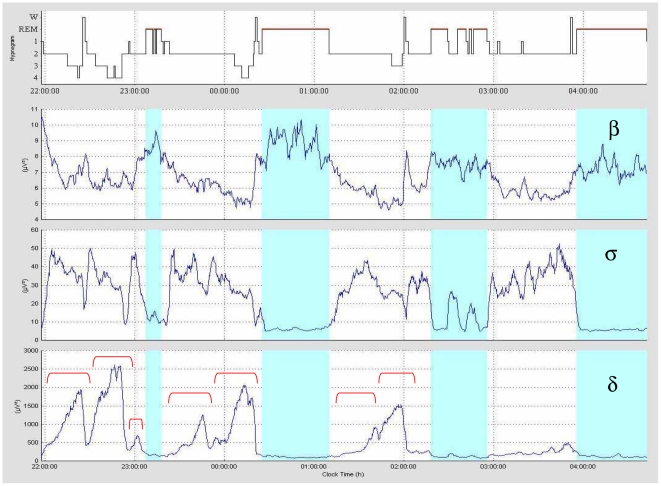
Sleep structure across NREM-REM sleep cycles 1 to 4 for a typical subject. Spectral power time-course data in the major frequency bands: beta (β) 18–25 Hz; sigma (σ) 12–15 Hz and delta (δ) 1–4 Hz, together with the corresponding hypnogram in the uppermost panel. W = wake; REM = REM sleep (shaded blue); 1, 2, 3, 4 = NREM sleep stages. The total duration of the time-courses is 6 h 50 min with the hours indicated by vertical dashed lines. Note the repeated alternations between going *towards* deep sleep (delta rising) and going *away* from deep sleep (delta falling) in each NREM episode. This is best visualised on the delta panel. *Towards*-and-*away* cycles (indicated by a red bracket) constitute the basic building blocks of the NREM episode.

**Figure 2:**
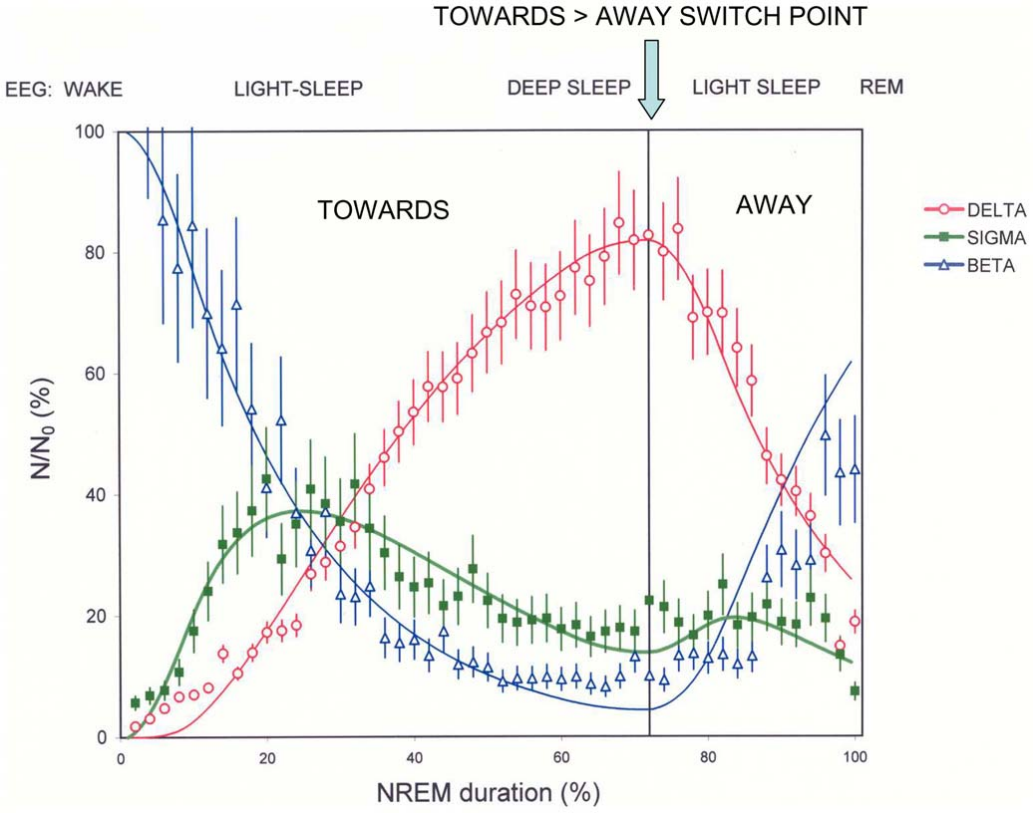
Power time-courses for beta, sigma and delta in NREM episode 1. Data are averaged over six healthy subjects having a single delta peak in NREM 1; error bars represent the standard error on the mean (adapted from figures 3a and 3b in Merica and Fortune [2]). The relation between the shapes of the time-course curves in the move *towards* deep sleep displays the distinct pattern on which the NTP model is based: beta power drops exponentially, delta power rises in an S-curve and sigma power reaches its maximum while delta is still rising. The number of neurons (N) in each mode beta, sigma and delta, is expressed as a percentage of the number of neurons in beta mode (N_0_) at the start of the NREM episode. N_0_ is the fixed-size of the generating population of the NTP model. The model fits the data well: overall goodness of fit as measured by the coefficient of determination R^2^ (%) = 92.6 (R^2^ delta = 97.8, R^2^ sigma = 82.0, R^2^ beta = 90.3).

Sleep is a complex behaviour under the control of several inter-related processes: a circadian process which determines periods of sleep propensity over 24 h; an ultradian process which is responsible for the architecture of the sleep period with its regular cyclic alternation of the two major sleep states, non-rapid eye movement (NREM) and rapid eye movement (REM) sleep; and dynamic processes which determine the temporal structure within these states. Governing each of these processes there is the homeostatic process which reflects the prior history of sleep and wake, and hence sleep pressure. It is precisely the finer structure within NREM, i.e. the temporal progression of the electroencephalogram (EEG), which has interested us for many years. This is a domain which has been somewhat neglected by others despite the fact that it shows *how* we sleep and has some fascinating aspects to it. In each ultradian cycle, EEG-defined states of vigilance succeed each other on the average in a simple and well-defined manner: wake to light sleep to deep slow wave sleep then back to light sleep before entry into REM sleep or wake. But in the main this simplicity holds only when data are averaged over several subjects [1], [2]. Individual subject data, however, often exhibit a more complex and revealing behaviour: Using the time-courses of spectral power in the sleep EEG, we showed [3] that there are in a typical first NREM episode, several moves *towards* and *away* from deep sleep (Figure 1), in each of which there is an identical distinct pattern linking the major frequency bands beta, sigma and delta (Figure 2). The essential of this pattern is that in the move *towards* deep sleep beta power drops exponentially, delta power rises in an S-curve and sigma power peaks while delta is still rising. This remarkable pattern repeatedly characterising the dynamic structure within NREM imperatively called for an explanation and this was given by our neuronal transition probability (NTP) model [2], [3].

To summarise briefly the lead-up to this model and the model itself, we note that much has been learnt in the recent past on the basic neuronal mechanisms involved in the control of the states of waking, NREM and REM sleep. This includes determining the sites of origin; mapping the widespread projections of the contained neuronal population to different cerebral structures; establishing the involvement of specific neurotransmitters and their actions on specific neurons; and unravelling the mechanisms underlying defined EEG rhythms [4]–[12]. All have contributed to a better understanding of the mechanisms that underlie the sleeping brain, making it possible to construct an increasingly clear picture of the permanent dialogue between cortical and sub-cortical structures giving rise to the electrical activity that characterises the different sleep states. However, relating these findings to an understanding of the temporal progression of EEG activity across the ultradian cycle is a challenge that has received little attention. A theoretical approach involving mathematical modelling offers a synthesis of the data in analytic terms, and thereby a possible route to understanding the cellular substrates underlying EEG dynamic structure. Over the years a number of very useful sleep models have been developed [13]–[24]. Reference [25] gives a comprehensive overview. None, however, address the crucial issue of explaining the temporal progression of the EEG over the entire sleep period. In this sense they do not answer the question of *how* we sleep. The NTP model [2], [3] is, to the best of our knowledge, the only model that does. It gives a coherent explanation for the relationship between the temporal progression of spectral power in the three major frequency bands of the NREM sleep EEG and thereby proposes a neurophysiological solution to the link between sub-cortical activities and EEG dynamics.

**Figure 3:**
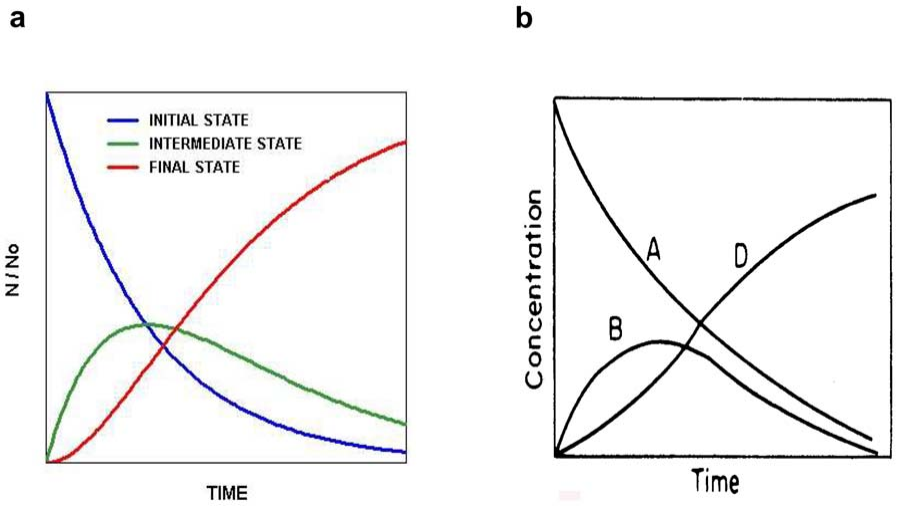
Three-stage cascade processes involving stochastic transitions in the hard sciences. (a) Physics: a 3-element cascade radioactive decay (adapted from Fermi Lectures: Chicago University press; 1950). This process starts at time = 0 with a fixed size population of radioactive atoms all in an identical initial state, where each atom has the same probability of transitioning to an intermediate radioactive state. Atoms arriving in that intermediate state are immediately subject to a probability of transitioning to the final stable state. The intermediate state reaches a maximum, when the number of atoms/unit time entering it, is the same as the number leaving it. Using the 2 probability parameters one can calculate the time courses of the relative number of atoms in each state. An important factor which simplifies the mathematics is that individual transitions are of very short duration relative to the duration of the population transition. (b) Chemistry: a 3-stage consecutive reaction of solutes within a solvent (adapted from Chemical Engineers Handbook (1973) with kind permission of the publisher). Gives the concentration-time profile for consecutive reaction A→B→D.

The conception of the model was prompted by the striking visual similarity between the pervasive EEG temporal pattern described above (Figure 2) and similar patterns already mathematically modelled in the hard sciences, suggesting that the EEG pattern could well be modelled by the same mathematics. The idea was encouraged by the fact that findings at the neuronal level accorded well with the conceptual basis of the hard science model (see below). In physics [26] the pattern (Figure 3a) describes a 3-element cascade radioactive decay. In chemistry [27] the same pattern (Figure 3b) describes a 3-stage consecutive reaction within a solvent. Postulating that the mathematical law governing cascade radioactive decay or chemical series reaction, also governs EEG spectral power time-course pattern, we adopted this analogy as premise for our model of NREM structure: where instead of atoms or molecules we have neurons; and instead of radioactive states or chemical solutes we have firing-rate frequencies. This mathematical law states that transitions from a given state to the next take place at a rate proportional to the amount of that state present and to the transition probability. This is the basis of the familiar law of exponential decline – a ubiquitous law, often seen in physiology. Exponential decline formulae, when applied to each level of a three-state cascading process produce the observed time-course curves. We therefore hypothesised that the NREM temporal pattern could be described as a cascade of neuronal firing-rate transitions corresponding to beta→sigma→delta in the sleep deepening (*towards*) phase, and an inverse cascade of transitions (delta→sigma→beta) in the sleep lightening (*away*) phase, taking as starting values for beta, sigma and delta the end values of the previous phase. Using this simple law, we found that the model is capable of simulating with remarkable precision, the shape of the time courses in both the *towards* and the *away* phases (Figure 2). To the best of our knowledge, no other model predicts how and when the two sigma peaks occur, while delta is rising and while delta is falling. We showed [3] that no matter how complex the structure, the model fits well the EEG data for the first NREM episode rich in slow wave sleep. It has only one premise and only one free parameter, a four-element probability vector P governing the shapes of the *towards* and the *away* time-course curves.

Not only does the model fit the spectral power time-course data, but the assumptions implicit in it - drawn from our analogy with the hard science models - also fit in with cellular data. At the cellular level the model makes several testable assumptions: (a) it postulates that there exists a fixed-size population of neurons that generates the spectral power time-courses. Cellular results [5], [10], [11], [28] provide substantial evidence suggesting that cholinergic and other thalamically projecting brainstem activating neurons constitute this generating population. We deduce this from evidence that changes in the discharge pattern of these neurons modulate the oscillatory mode of thalamocortical cells. Illustrating this brainstem modulation, cellular data show [28] that there is a direct parallelism between brainstem firing rate and EEG activity, where the appearance of each spindle is concurrent with a marked decrease in firing rate (Figure S1-a). This is also shown in the parallelism between the U-shaped time-course of the mean firing rate of a thalamically projecting brainstem activating neuron and the sleep state progression in the move *towards* and *away* from deep sleep. (See Figure 4.15 in [29]). If the brainstem activating system is modulating sleep state progression, then it is reasonable to suppose that it is *a fortiori* modulating the beta, sigma and delta power time-courses. Several EEG results also support the concept of brainstem control: for example, we found [30] that the shapes, timing and relationships of the observed beta, sigma and delta power time-courses are identical at all cortical sites. So there is a strong case for assuming that a template time-course pattern is generated in the brainstem activating system and is propagated to the thalamus and thence to all sites at the cortex. Video S1 provides a visualisation of what we suppose, on the basis of NTP model, is happening at the brainstem generating population during the course of a typical NREM *towards-away* cycle – and how it parallels progression at the EEG. (b) The model postulates that the firing rate mode transitions in individual neurons are ‘instantaneous’ relative to the time scale of the NREM episode. There is evidence from Steriade's group [28] (Figure S1-b) and from unit measurement data [31] on the firing rate mode change in the sleep-promoting neurons located in the ventrolateral preoptic nucleus (VLPO) (Figure S2) that supports this assumption. (c) NTP considers NREM temporal progression as an alternation of two steady dynamic states – *going towards* and *going away* from deep sleep. Only in this way is it possible to postulate the physiological reason for the relationship between the spectral power time-courses of beta, sigma and delta. The NTP model equations govern the shapes of the time-course curves within each of the two states, but do not give the timing of the alternation between these states. The NTP model therefore postulates the existence of an external flip-flop switch to perform this timing [2]. The immediate unanswered question is: Where is this control switch located?

In the light of all these arguments in favour of the NTP model, our aim here is try to complete the picture in two ways (a) determine where the master switch is located and how it exerts its control; and (b) apply the NTP model fitting to the later cycles to determine how the model can accommodate the EEG changes induced by the lowering of sleep pressure across the night. In our results we show that the NTP model can successfully fit the later NREM episodes and that the suprachiasmatic nucleus (SCN) is a prime candidate for the control of the timing of the repeated switching between *towards* and *away* alternations within each episode. This supports our view of a sleep dynamic structure under the *dual* control of the SCN and the NTP model: the state-initiating timer is at the SCN and it alone provides the start and stop times of the *towards*/*away* states; the detailed shapes of all time-course curves within these states are governed only by the NTP model equations.

## Results

### Localizing the Flip-flop Towards-Away Switch Inherent in the NTP Model

The strength of the NTP model since its inception has lain in the solidity of the tenets on which it is based, and on the good agreement between theory and data. This strength is independent of the location of the master flip-flop switch. Nevertheless, finding this location would surely put the model on an even firmer footing by reinforcing its physiological basis. In fact, there are several reasons to suppose that the SCN provides the flip-flop switching action postulated more than a decade ago by the NTP model: It is widely accepted that the SCN is the source of the circadian signal for the occurrence and consolidation of sleep and wakefulness; there exists anatomical evidence that sleep-promoting and wake-promoting neurons receive input both directly and indirectly from the SCN [16], [32]–[34]; and more to the point, recent studies [35], [36] provide evidence that suggests to us the existence at the SCN of the flip-flop switch we are looking for. Deboer and colleagues [35] simultaneously recorded EEG activity and electrical neuronal activity in the SCN in un-anesthetized rats and showed that changes in vigilance states are paralleled by changes in SCN activity, irrespective of circadian phase. They showed that within NREM there is a high and statistically significant negative correlation between minute by minute changes in the firing rate of SCN neurons and slow wave activity (SWA). Similar findings have been reported in humans [37]. The Deboer et al. findings, backed by anatomical evidence showing that there are direct cholinergic [38] and serotonergic [39] projections from the arousal centres to the SCN, allow them to conclude that the SCN is continuously changing its level of activity under the influence of feedback from the sleep centres. Here – in line with the core ideas of our NTP model and with the consensus that SCN controls wake/sleep alternations and is therefore a causal agent with regard to the vigilance states - we propose that it is the sleep centres that are being controlled by the SCN. The SCN is continuously changing its level of activity under the influence of feedback from several sleep-related sources integrated via the dorsomedial nucleus of the hypothalamus [33]. These sources include homeostatic drives (sleep pressures, hunger etc.) and environmental conditions (light, sound, temperature, social stimuli etc.). Since the SCN is acknowledged to be the master time-keeper of the brain, we infer that it uses its continuously changing activity to create a flip-flop timing control mechanism functioning similarly to standard electronic circuits converting analogue data into digital two-state switches using a preset threshold. We therefore examined the Deboer et al. data in this light.

**Figure 4:**
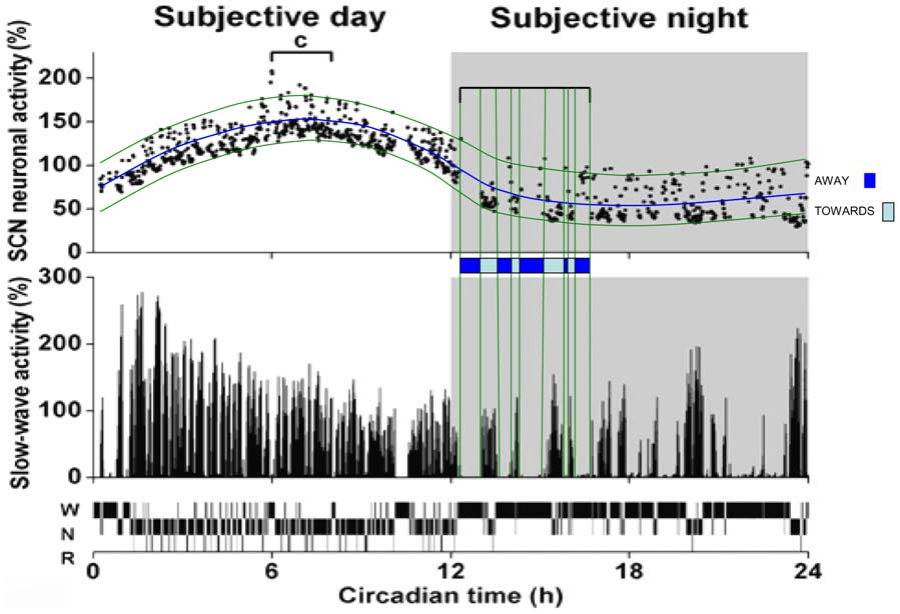
Vigilance states, slow-wave activity and SCN neuronal activity: 24 hour recording. SWA (EEG power density 1–4 Hz) and SCN neuronal activity are plotted as a percentage of the mean activity during NREM sleep over 24 h (each data point is the mean of 6 10-s epochs). The grey background indicates the subjective night (dark period) characterised by a lower level of SCN activity where the nocturnal rat is mostly active but exhibits short bouts of sleep. SWA shows high values during NREM sleep (N) and low values during REM sleep (R) and waking (W), adapted from Deboer et al. [35] with the kind permission of the publisher. The two green curves we added on the SCN activity figure enclose most of the data points. We also added about half way between these green lines a blue threshold line which separates SCN activity into two zones: one corresponding to a WAKE or REM level where SWA is about zero and the other to a NREM level where SWA is high. These zones correspond to the ‘*Away*’ and ‘*Towards*’ phases of the NTP model. The blue colour-coded bar above the SWA figure show the instants of transition of the SCN data across the threshold line, in either the upward or the downward direction. The inserted vertical green alignment lines facilitate the visualisation of simultaneity between transition events in the SCN activity and SWA.

**Figure 5:**
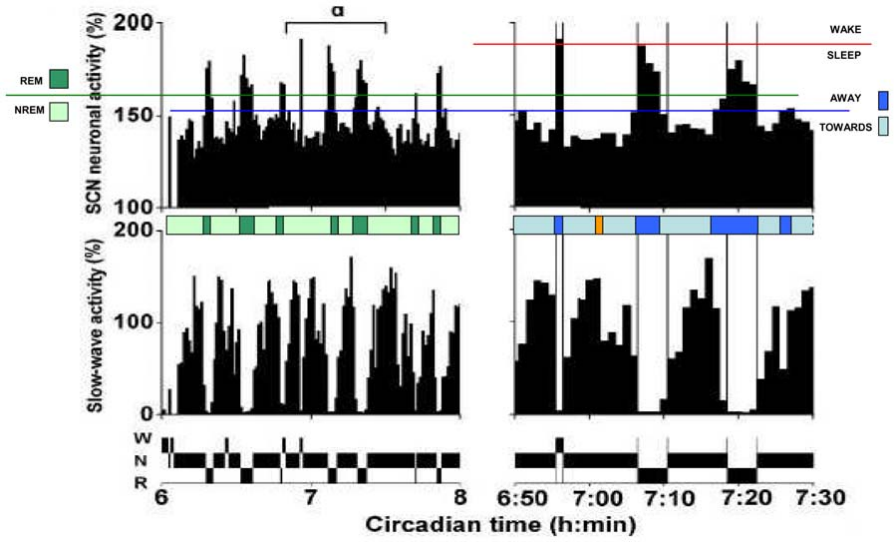
Vigilance states, slow-wave activity and SCN neuronal activity. The left hand panels show an expansion of the data in Figure 4 (labelled c), representing 2 hours around the circadian peak. Right hand panels are an expansion of a portion of the recordings in the left hand panels (labelled a). Vertical lines on the right hand panels indicate vigilance state transitions, adapted from Deboer et al. [35] with the kind permission of the publisher. We have added a horizontal blue line on the SCN activity figure, above which SWA is falling rapidly to zero and below which SWA is rising slowly. These zones correspond to the ‘*Away*’ and ‘*Towards*’ phases of the NTP model. The blue colour-coded bar above the SWA figure shows the start and stop times of each phase. The orange rectangle on this bar indicates the position of an away phase of the SWA where the SCN data do not cross the threshold line as would be expected. The green colour-coded bar added above the SWA figure on the left hand panel, based on the green line threshold at about 160% on the SCN activity panel, show the SCN potential to control NREM-REM state transitions.

The results shown in colour in Figures 4 and 5 are essentially our interpretation of the Deboer et al. data [35]. Figure 4 shows the relationship between vigilance states and SCN neuronal activity over the full circadian cycle. We have added on their SCN panel green lines enclosing most of the data points in order to show that the variation of SCN neuronal activity at any time is almost constant over the entire 24 hours despite the substantial difference in mean levels during the dark and light periods. The strong negative correlation between SCN and SWA reported by Deboer et al. is clearly brought out throughout the circadian cycle by the separation of the SCN data into two perceptible zones: one corresponding to a WAKE or REM level where SWA is about zero and the other to a NREM level where SWA is high. We therefore added about half way between the green lines a blue threshold line which separates these zones and we then closely examined the temporal alignments between the SCN activity threshold crossovers and the SWA transition points in the first part of the subjective night and in the middle of the subjective day.

We focus first on the early part of the night period, indicated by a horizontal bracket, where the blue threshold line curves downwards from ∼90% to a quasi-flat nadir at ∼50% on their SCN activity scale. To facilitate our analysis we inserted vertical green alignment lines (see methods) showing the instants of transition of the SCN data across the threshold line, which are summarised in the colour-coded bar. Following these alignment lines downward it is clear that all 10 of these instants coincide with the start or stop of each *towards* or *away* interval of the SWA - strongly suggesting by this 100% correlation between SCN trigger and SWA reaction that the SCN is providing the flip-flop control of the *towards* and *away* transition points inherent in the NTP model. Note that in the interval studied, the SCN is effectively timing sleep/wake transitions since there is very little REM sleep present. This result concurs with the general consensus that sleep/wake transitions are under SCN control – with the SCN acting as causal agent.

Turning now to the day period where the animal is mainly sleeping, we carried out a similar analysis on the data around the circadian peak, labelled ‘c’ in Figure 4. An expansion of these data is shown in Figure 5 (left-hand panels). Right hand panels are a further expansion of a portion of the recordings in the left hand panels (labelled ‘a’). Focusing on this right hand panel, we have added to Figure 5 part of the curved blue threshold line of Figure 4 which for this ‘a’ interval is situated at about 150% on their SCN activity scale, and which can be taken here to be straight and horizontal. Again, we constructed the transition points of the SCN data across the blue threshold line with the results given in the blue colour-coded bar: there are 9 threshold crossover points compared with 11 *towards* and *away* transitions apparent in the SWA data. The one case where the SCN does not appear to trigger the *away* phase, concerns the 2^nd^ *away* phase in the first NREM episode of the right hand panel, at the time interval indicated by the orange-coloured rectangle on the blue colour-coded bar. This we attribute to the effect of binning the data (see methods). If the binning were less coarse, we strongly suspect that a sharp peak in SCN activity would be seen to occur at the position of the orange-coloured rectangle. Following the lines of the colour-coded bar downward, it is then clear that at least 9 out of the total of 11 SWA transition points are successfully predicted by the SCN – 11 out of 11 if there actually is a sharp peak of SCN activity masked by binning. Nonetheless 9 out of 11, corresponding to 83% correlation between SCN trigger and SWA reaction, is a convincing score. On the basis of our findings in the beginning of the night and the middle of the day we propose that the blue threshold line and the threshold crossover instants can be interpreted as defining the timing of an SCN-located flip-flop controller, switching the steady probability states of the NTP model between inciting a move *towards* deep sleep - and inciting a move *away*. We complement these results by noting that if we add another threshold line coloured green at a level of about 160% (Figure 5 left hand panel) and analyse the data in the same way, we get the green colour-coded bar and we see how the SCN also has the potential to switch the NREM-REM state transitions. In fact, it does so with a success rate of 100% (14 out of 14) over the entire two hours studied. In fact, of all 35 transition points we have examined in wake/sleep, NREM/REM, and *towards/away*, the SCN has predicted correctly the instant of SWA transition in 33 of them. In short, our results: concur with the consensus that the SCN actively promotes wake/sleep alternations; they strongly suggest the SCN also directs the timing of NREM-REM alternations; and finally they strongly suggest that the SCN also times the switching within NREM of the two steady dynamic states - *going towards* and *going away* from deep sleep.

**Figure 6:**
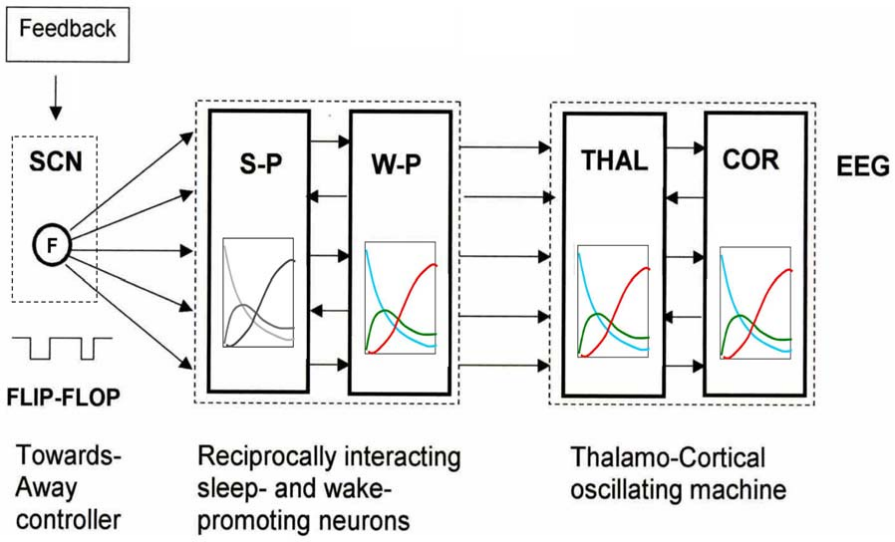
SCN to EEG: a graphic summary of the NTP model for NREM sleep structure. Dual control by the SCN switch and the NTP model equations: the SCN, the acknowledged time-keeper of the brain, changes its level of activity under the influence of feedback from several sources in particular homeostatic drive, it then uses these changes to create a flip-flop (F) timing control that switches downstream action between the two dynamic states going *towards* and going *away* from deep sleep i.e. between P_1_P_2_ and P_3_P_4_. These instructions are conveyed to the sleep-promoting (S-P) and wake-promoting (W-P) population pair which by reciprocal interaction are locked in anti-phase, like the 2 ends of a see-saw. Here the NTP equations take over and determine, as generating population at the brainstem, the template pattern of firing-rate time-courses of the brainstem activating neurons. The template propagates downstream and modulates thalamic output. Thalamocortical networks then form the complex wave sequences observed on the EEG, while following the power time-course pattern dictated by the brainstem. For simplicity, only the first *towards* phase of the template pattern is shown in the inset boxes. The box for the S-P population is in grayscale to indicate that the vertical axis is different from that of the colored patterns following: it indicates the fraction of neurons in each equivalent firing-rate mode (β, σ and δ) corresponding to the cascade (relatively silent→moderate firing-rate→fast firing-rate) rather than the cascade (fast firing-rate→moderate firing-rate→relatively silent) in the colored patterns. Thus the mean firing-rate in the S-P population (grayscale) progressively increases with time, while in the W-P population (colour) it decreases.

We can now state that the original NTP assumptions concerning: flip-flop timer control; the relative instantaneity of individual neuronal transitions; and the existence of a generating population, are all to a large extent supported by data. Based on this and findings at the neuronal level [[31], [40]–[55], see Text S1] Figure 6 gives an overview of the chain of events from SCN to EEG embodying our global model for the structure of NREM.

### NTP Model Fitting Applied to the Later Cycles

Our earlier results on the good fit of the NTP model to the data in the first NREM episode [3] together with the awareness that as sleep pressure dissipates across the night both delta power and sigma power undergo significant changes, incited us to further test the model by determining how it can accommodate these changes.

Inspection of all night sleep as measured by standard sleep stage variables indicated that the quality of sleep for the subjects studied is well within the normal range. Sleep latency, defined as the interval between lights out and the first occurrence of stage 2 sleep, was 16.5±2.2 (SEM) minutes. The sleep efficiency index, defined as the ratio of total sleep time (excluding waking intervals) to total sleep period (including waking intervals) was 0.98±0.004.

**Figure 7:**
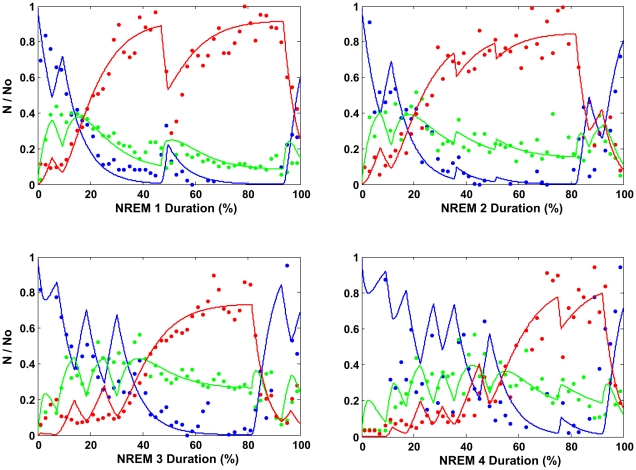
The NTP model fit to the data of representative NREM episodes 1 to 4. Data is represented by filled circles: beta (blue), sigma (green) and delta (red). The time scale is given as a percentage of the total duration of the given NREM episode. The number of neurons (N) in each mode beta, sigma and delta, is expressed as a percentage of the number of neurons in beta mode (N_0_) at the start of the NREM episode. The model with the fixed probability set fits well all four NREM episodes: NREM 1 with 3 *towards*-and-*away* (TA) cycles: overall goodness of fit as measured by R^2^ (%) = 81.2, (R^2^ delta = 88.6, R^2^ sigma = 63.6, R^2^ beta = 84.7). NREM 2: 5 TA cycles; overall R^2^ = 75.5, (R^2^ delta = 89.2, R^2^ sigma = 48.6, R^2^ beta = 76.2). NREM 3: 5 TA cycles; overall R^2^ = 75.7, (R^2^ delta = 90.8, R^2^ sigma = 52.6, R^2^ beta = 70.3). NREM 4: 7 TA cycles; overall R^2^ = 63.1, (R^2^ delta = 86.2, R^2^ sigma = 46.1, R^2^ beta = 42.4).

Using the probability vector P [0.13, 0.131, 0.2, 0.6] established in the methods section, Table 1 gives the R^2^ quality of fit values for each subject, showing in each NREM episode the proportion of the total variation in the delta, sigma and beta data curves that is explained by the model, as well as an arbitrary classification of R^2^ into 3 classes. In all cases the residuals were checked to be randomly distributed around zero. The results show the quality of fit to be very good in NREM 1 and good in NREMs 2 to 4. Repeated measures ANOVA results showed that there is a significant overnight effect on R^2^ across the four NREM episodes [*F* (3, 87) = 30.66, *P*<0.00005], with a significant overnight linear decline [*F* (1, 29) = 149.61, *P*<0.00005] that accounts for 94.4% of the variability across consecutive NREM episodes. Nonetheless, despite this decline, there can be no doubt as to the model's reliable performance throughout the night. Table 2 shows that in all 4 episodes the model fits the data with R^2^ very good to good in the majority of cases. The fit of the NTP model to the data of representative NREM episodes 1 to 4 is shown in Figure 7. The results of a detailed evaluation of the quality of fit for the 30 subjects showed an overall value for delta as very good throughout the night and for sigma and beta sufficient for the model to bring out their salient features (Table S1). The average goodness of fit R^2^ values over all subjects in NREMs 1 to 4 respectively, calculated from the Fisher z-transformed values, are 0.855, 0.802, 0.759, 0.592 for delta, 0.479, 0.367, 0.325, 0.356 for sigma and 0.672, 0.422, 0.396, 0.320 for beta.

**Table 1:** Fit of the NTP model to individual subject data across the night.

	NREM 1			NREM 2			NREM 3			NREM 4		
Subject	TA	R^2^	Quality	TA	R^2^	Quality	TA	R^2^	Quality	TA	R^2^	Quality
1	5	66.1	VG	4	74.0	VG	6	37.9	F	7	53.8	G
2	4	62.2	VG	5	35.4	F	7	39.6	F	7	37.8	F
3	5	64.2	VG	7	40.6	G	5	45.1	G	8	46.6	G
4	4	84.8	VG	8	33.7	F	4	60.7	VG	10	53.8	G
5	3	81.2	VG	7	67.0	VG	8	64.4	VG	6	34.5	F
6	7	72.3	VG	4	75.7	VG	4	33.3	F	5	58.6	G
7	6	65.7	VG	7	43.9	G	5	75.7	VG	6	37.7	F
8	6	67.4	VG	5	61.5	VG	8	30.9	F	7	43.1	G
9	5	65.0	VG	7	63.7	VG	9	62.0	VG	8	40.7	G
10	2	76.0	VG	6	47.9	G	5	65.2	VG	7	19.0	F
11	7	63.5	VG	5	75.5	VG	10	49.7	G	7	49.1	G
12	6	61.5	VG	8	42.7	G	7	32.9	F	8	41.8	G
13	5	56.5	G	7	56.3	G	6	52.9	G	8	51.9	G
14	7	58.5	G	6	66.2	VG	6	39.5	F	6	35.4	F
15	7	57.4	G	7	57.8	G	9	71.4	VG	7	41.8	G
16	4	77.5	VG	8	57.9	G	7	50.6	G	7	63.1	VG
17	5	74.8	VG	7	61.1	VG	6	43.3	G	7	56.2	G
18	4	73.2	VG	9	51.9	G	9	38.1	F	7	60.9	VG
19	3	70.5	VG	6	54.9	G	6	55.2	G	3	28.7	F
20	5	82.0	VG	8	45.8	G	7	55.7	G	5	26.8	F
21	6	63.3	VG	5	66.3	VG	7	26.6	F	8	53.0	G
22	8	73.0	VG	7	46.9	G	6	57.4	G	7	37.0	F
23	7	66.1	VG	7	63.3	VG	7	47.7	G	7	39.1	F
24	4	70.7	VG	6	45.5	G	8	65.1	VG	6	28.9	F
25	5	74.9	VG	8	48.4	G	6	61.4	VG	10	40.2	G
26	6	71.2	VG	7	55.1	G	7	58.8	G	7	33.5	F
27	5	83.3	VG	9	59.5	G	7	53.6	G	7	36.0	F
28	5	58.7	G	7	48.0	G	8	47.1	G	9	43.7	G
29	5	66.9	VG	8	65.8	VG	7	65.2	VG	8	47.3	G
30	5	63.7	VG	7	53.9	G	8	43.8	G	8	34.3	F
**Mean**	**5.2**	**69.9**	**VG**	**6.7**	**56.4**	**G**	**6.8**	**51.9**	**G**	**7.1**	**42.8**	**G**

T/A = Number of towards and away cycles within the NREM episode; R^2^ = Coefficient of determination expressed in %, measures the overall goodness of fit of the model to the data; VG = Very Good fit, R^2^≥60%; G = Good fit, R^2^≥40 and <60%; F = Fair fit, R^2^≥19 and <40%. Mean R^2^ values calculated using the Fisher z-transformed values.

**Table 2:** Percentage of NREM episodes in each quality of fit category.

Quality of fit	R^2^ %	NREM 1	NREM 2	NREM 3	NREM 4
Very good	≥60 and ≤85	86.7	36.7	30.0	6.7
Good	≥40 and <60	13.3	56.7	43.3	50.0
Fairly good	≥19 and <40	0	6.6	26.7	43.3

In addition, Table 1 gives the number of *towards and away* (TA) cycles within each NREM episode. It is our contention that these cycles – each with its alternation of the two steady dynamic states: a move *towards* followed by a move *away* from deep sleep - constitute the basic building blocks of the NREM episode. The times of switchover between the two dynamic states are an important parameter in the model since they determine the structure of each episode. The results showed an increase in the number of TA cycles per NREM across consecutive episodes. Repeated measures ANOVA results showed that this rise is significant [*F* (3, 87) = 12.28, *P*<0.00005], with significant overnight linear [*F* (1, 29) = 33.15, *P*<0.00005] and quadratic [*F* (1, 29) = 6.59, *P*<0.02] trends that respectively account for 76.1% and 18.1% of the variability across consecutive NREM episodes.

## Discussion

**Figure 8:**
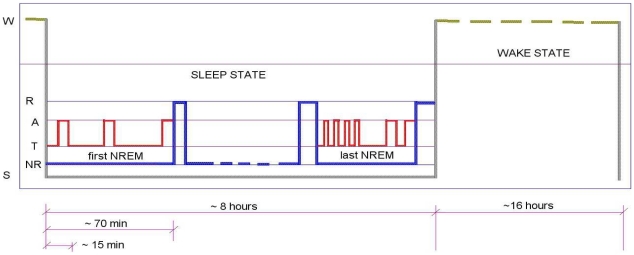
Overview of SCN sleep timers. As we see it, the SCN, influenced by feedback from several sources in particular homeostatic drives, has all the information necessary to provide sleep timing flip-flops not only for wake-sleep (W-S) and NREM-REM (NR-R) transitions but also for the *towards* (T) and *away* (A) switchovers within NREM. Thus there would appear to be a progressive penetration of SCN control into all layers of the complex behaviour that is sleep: in the outer layer a control of the circadian process (wake-sleep alternation), in the next layer a control of the ultradian process (NREM-REM alternation) and in the next deeper layer a control of the process responsible for NREM sleep structure (*towards* and *away* alternation within NREM).

The present study examines 2 issues related to the NTP model: where is located the master-switch inherent in the model, and does the model fit well all NREM episodes. Taking each in turn:

Our present results on the localization of the master switch strongly suggest that the SCN is the final long sought for link in the chain of events that give rise to the dynamic progression within NREM sleep EEG as described by the NTP model. The role of the SCN as a master pacemaker, timing the occurrence of sleep and wake has been recognized for some time and our interpretation of Deboer's data, with the SCN as causal agent, is fully in accordance with this role (Figure 4). Moreover recent studies suggest that this timing function may be extended to the control of NREM-REM alternation: In addition to data that show that REM sleep expression is governed by circadian factors [56], there is evidence that suggests a role for the SCN in controlling sleep architecture by the active promotion of REM sleep [57], [58]. Our results (Figure 5) support this view and extend this role further by strongly suggesting that the SCN also controls the timing of the *towards* and *away* phases of NREM sleep structure. An overview of the SCN sleep timers, as we see it, is schematically represented in Figure 8, and sheds an exciting new light on the possibly much wider involvement of the SCN in the control of sleep. The timing function of the SCN finds significant support in SCN ablation data [34]: essentially, the excision of the SCN has the principal effect of introducing serious disorder in the timing control of the vigilance states. SCN ablation eliminates sleep-wake circadian rhythms without disrupting intrinsic sleep need, such that the 24 hour day is randomly organised into bouts of sleep and bouts of wake with no clear division between day and night behaviour. This is just what we would expect under our hypothesis of SCN control of sleep timing: take away that control and timing chaos ensues. Our proposal that the SCN controls vigilance state changes is compatible with Saper et al. [33] but differs fundamentally from that of Deboer et al. who, on the basis of deprivation experiments [35], [36] conclude that changes in SCN neuronal activity are caused by changes in vigilance states. It is our view that the deprivation experiments do not allow this conclusion on causality to be drawn since they overlook the contribution of important confounding factors (homeostatic pressure and/or environmental perturbation) which may be the root cause of the observed changes in SCN activity and consequently in the EEG slow wave activity.

As concerns NTP model fitting in the later cycles, the results clearly show that the model can accurately reproduce the very particular dynamic relationship between power in the major frequency bands in all four NREM episodes. The tremendous simplification that comes with the fact that it does so with a fixed set of probabilities for all episodes comes as somewhat of a surprise since we had speculated [2] that the model could in principle simulate the main characteristics that distinguish the later NREM episodes from the early ones simply by lowering the sigma-to-delta and increasing the delta-to-sigma transition probability values. In our speculation, however, we omitted to attribute sufficient importance to the variation in the number of *towards* and *away* switchovers per NREM, which now appears to be a crucial aspect of NREM structure. Thus we conclude that the EEG changes induced by the lowering of sleep pressure in the later cycles are very probably due to this and not due to local changes in transition probabilities. This emphasis on the probabilities – and their possible role as a “constant of nature” - raises again the question of what cellular mechanism could give rise to the probabilistic control of the firing rate modes of the brainstem activating neurons at the root of the NTP model. Where do the probabilities come from? We have previously suggested [3] that on the supposition that the firing rates are modulated by neurotransmitter release probabilities then the NTP probabilities could perhaps be related to these release probabilities at the synapses linking the *towards* and *away* switch to the sleep-promoting neuronal population. This proposition finds support in recent literature on synaptic transmission [59].

The results show that the model can accommodate the EEG changes induced by the lowering of sleep pressure across the night, by increasing the number of *towards* and *away* switchovers per NREM across consecutive episodes. In the light of the NTP theory, the increased number of moves away from deep sleep in the first half of the later episodes repeatedly interrupts a smooth beta→sigma→delta cascade hence hampering the delta power build-up. Thus our results suggest that it is the instability instilled by the dissipation of sleep pressure that is responsible for the changes in both SWA and spindle frequency activity that characterise the later NREM episodes. Situating this result in the light of our global model for sleep structure summarised in Figure 6, we propose that as the night progresses the sleep state gets nearer to the sleep-wake pressure balance point causing increased *towards* and *away* switching by the SCN due to the increasing homeostatic instability fed back to it. Our interpretation is similar to that proposed by Saper and colleagues [15], [33] to describe the functioning of their sleep-wake flip-flop switch, confined to transitions from wakefulness to sleep (sleep onset) and from sleep to wakefulness (sleep offset). There is however a fundamental difference in location of the switch. Saper et al. advocate that the flip-flop neuronal circuit forming their switch is composed of the mutually inhibitory sleep promoting neurons in the VLPO on one side and the wake promoting neurons in the major arousal centres on the other. Increased activity in either of the competing sides “causes the switch to flip state” and that when “either side is weakened, homeostatic forces cause the switch to ride closer to its transition point” resulting in increased transitions [33]. We on the other hand, backed by data, advocate that it is the SCN operating on feedback from several sources in particular homeostatic drives, which provides the switching action between wakefulness and sleep and between going *towards* and going *away* from deep sleep throughout the sleep period. This standpoint finds support in experimental evidence showing that the SCN can attenuate sleep through the inhibition of the VLPO sleep-promoting neurons [60].

Finally, we attribute the somewhat lesser quality of fit in the later NREM episodes, characterised by less consolidated sleep, to a noisier EEG signal. To try and explain the increased jitter in the EEG signal in physiological terms, we refer to recent findings on the changes in cortical firing in relation to sleep pressure [61]. Intracellular recordings have shown that during NREM sleep virtually all cortical neurons alternate between a depolarized ‘up’ or active state, and a hyperpolarized ‘down’ or silent state. This cortically generated slow oscillation (<1 Hz) occurs more or less synchronously across many neurons [53] and is responsible for triggering, shaping and synchronizing thalamically generated rhythms which otherwise would not have sufficient amplitude to be effective at the EEG level [49]–[51]. Recent findings, however, show that neuronal synchronization during the transition between ‘up’ and ‘down’ periods changes across the night. In the early NREM episodes, individual neurons in the population stop or resume firing in near synchrony whereas in the later episodes these transitions are much less synchronous [61]. This lack of synchrony could, in our view, give rise to a noisier and hence a more difficult to fit EEG signal.

To sum up, the proposed tandem control within NREM sleep by the SCN and by the NTP equations introduces a totally new way of looking at sleep dynamics. Both the NTP model and the SCN connection to it, encourage some fascinating avenues of research. They incite research at cellular level to verify the existence of the NTP template pattern in the brainstem by the analysis of firing rates in the modes corresponding to beta, sigma and delta oscillations, and to further investigate the cellular mechanisms that give rise to the probabilistic control of the firing rate modes at the root of the NTP theory. They also incite verification by dedicated experimentation of the SCN role as the controlling flip-flop timer in the NTP model, and an investigation of the mechanism by which the SCN fixes its continuously changing threshold levels to produce the observed switching between vigilance states. Finally it incites a systematic study of pathological sleep effects on the NTP parameters as an avenue to clinical applications of the theory.

In conclusion, our results provide unequivocal evidence that the NTP model fits all NREM episodes and they show how the SCN may be providing the timing signals for all sleep state switching including that required for the NTP model. The model has the unique merit of integrating neurophysiological data at the cellular level with detailed time-course data at the EEG level, leading to a global picture (Figure 6) for NREM structure all the way from the SCN to the EEG - determined only by SCN switching and NTP model probabilities.

## Materials and Methods

### Testing the hypothesis that the SCN controls NTP timing

We looked for evidence in support of the ability of the SCN to control the timing of the *towards* and *away* transitions in NREM. The acknowledged ability of the SCN to control the timing of sleep/wake transitions implies its ability to output two-state flip-flop instructions. This in turn implies that the SCN uses threshold control. We therefore examined the data in this light. In threshold control one asks at what instant does the SCN activity level cross a threshold line, in either the upward or the downward direction. The exact comportment of the analogue activity below or above the threshold is not relevant. The determination of these instants was done by carefully measuring simultaneity (temporal alignment) between transition events in the SCN and in the SWS activities. To do this with precision we have added vertical cursors and these we aligned to the original Deboer SCN data series. Provided the data were not too crowded or the time line was suitably expanded, the alignment could be done with precision. With these limitations, we estimated that we could locate the instants of SCN transitions across the threshold line, with a precision of about ± half a bin width (approximately 30 seconds) and these were summarised in colour-coded bars. Errors in establishing SWA transitions between the dynamic towards/away states are of similar magnitude. One minor error source is the binning effect, or resolution limitation. This can intervene for example if the bin limit falls on the middle of a sharp ∼1 bin-width peak in SCN activity (a sharp dip in SWA) then the level of SCN activity in the bins on either side of the peak would be much reduced relative to the true peak amplitude - and the SCN activity level would then not appear to reach the threshold line as expected from the SWA behaviour.

### Subjects

The study uses data from 30 healthy paid volunteers aged between 20 and 35 years (mean age 24.8±3.5 years) from whom written informed consent was obtained in accordance with local Ethical Committee requirements. Ethics approval for this retrospective study was obtained from the “Commission centrale d'éthique de la recherche sur l'être humain des HUG”. The subjects were selected randomly from our data bank of all-night drug-free sleep recordings carried out under controlled environmental conditions. Lights-off and lights-on were scheduled at 22:00 h and 07:00 h. The subjects were screened for good health and the absence of any sleep disturbances determined on the basis of a first night polysomnography that also served as a habituation night.

### Sleep EEG recording, Data extraction and Spectral analysis

All night sleep was recorded using three bipolar EEG derivations (F4-CZ, C4-T4, PZ-O2), one horizontal electrooculogram, one sub-mental electromyogram, an electrocardiogram and respiration (monitored by thermistors placed under the nostrils). Sleep stages were visually scored in 20-s epochs according to Rechtschaffen and Kales rules [62]. The EEG signals were high- and low-pass filtered (0.5 and 70 Hz) and digitised at a sampling rate of 256 Hz with a 12-bit resolution. Prior to analyses, the signals were subjected to an automatic 1-s resolution artefact detection routine using a background-dependent filter based on the root mean square amplitude of the signals. After visual validation, all epochs containing artefacts were coded as missing data so as to preserve time continuity. EEG power spectra in units of µV^2^ were computed by fast Fourier transform with a Hanning window for consecutive 4-s epochs giving a 0.25 Hz resolution over a frequency range 0.5–35 Hz. This range was divided into the three major frequency bands: delta (1–4 Hz), sigma (12–15 Hz) and beta (∼18–25 Hz). On the basis of our recent finding [63], the lower limit of the beta band was determined for each subject. The study was carried out on the F4-CZ EEG signal. The use of a single derivation is justified since we have shown that the shape and timing of the spectral power time-courses is quasi identical at all cortical sites [30]. The time courses of absolute power (µV^2^ per resolution time interval) were analysed separately in each NREM episode. The point of separation between REM and NREM episodes was determined using the 15 min combining rule for defining the end of a REM episode [64], [65]. No minimum REM sleep duration was required to define the start of a REM episode. The start of the first NREM was episode was set at sleep onset. The epoch that immediately follows the end of a REM episode gave the start for the following NREM episode. We retained the first four NREM episodes for study. Sleep staging, preliminary artefact rejection and spectral analysis were done using the PRANA software package from PHITOOLS Grenoble, France.

### Fitting of the model

Model fitting was carried out on individual subject data so as to preserve the true NREM structure dynamics. Given the wide inter-subject variation in the timing of the moves *towards* and *away* from deep sleep, the use of averaged data would conceal the structure of the individual time-courses constituting the average.

NREM episode duration was normalized to 100% and the average power in each frequency band calculated for each 2% bin. Initially delta was normalized to the maximum data value. Then initial values for the times of switchovers between moving *towards* (hyperpolarizing phase) and moving *away* (depolarizing phase) from deep sleep were determined by peak and trough detection using three-point moving average smoothing of the delta time-course. The NTP model was then fitted to each episode in turn with starting values for each *towards* and *away* phase taken as equal to the end values of the previous phase. The fitting parameters were the beta, sigma and delta power normalization factors common throughout the episode; a 4-element transition probability vector P whose components are P_βσ_, P_σδ_, P_δσ_ and P_σβ_, common to all *towards* and *away* cycles in the episode; and the switchover times. We found that optimising the transition probability values for individual NREM episodes did not significantly improve the results, so the model was considerably simplified by fitting all 4 NREM episodes using a common transition probability set: P = [0.13, 0.131, 0.2, 0.6] per 1% of the NREM duration. Delta power is more precisely measured than either sigma or beta, so delta was fitted first and the delta parameters fine–tuned using the coefficient of determination R^2^ as a goodness-of-fit criterion. The vertical scaling factors for the sigma and beta curves were then optimised, using as initial values the switchover vector already determined for delta. To fit beta, a bias of about 2 µV^2^, corresponding to a constant background beta component present throughout sleep, was subtracted from the data. Using the Fisher z-transformation, an overall R^2^ was computed for the three curves together in each NREM episode. The episode R^2^ values were then classified into three categories: Very good fit (R^2^≥60%), good fit (R^2^≥40 and <60%), fairly good fit (R^2^≥19 and <40%).

### Statistical analysis

The time of night effect on the quality of fit and on the number of *towards* and *away* cycles per NREM were tested by repeated measures analysis of variance (ANOVA). To protect against violation of the required compound symmetry assumption, the Huynh-Feldt adjustment of the probability was used and the result cross-checked by multivariate repeated measures analysis that does not require compound symmetry. Trends across the night were tested using single degree of freedom polynomial contrasts.

### Model Equations

The simultaneous pattern of change in the beta, sigma and delta power time-course curves in the sleep deepening (*towards*) phase is similar to that produced by a 3-element radioactive decay chain and is expressed mathematically by the same formulae [26]. The fundamental idea behind both processes is that the rate of decrease of a quantity N is proportional to the quantity itself: dN/dT = −pN where N = quantity at time T; p = transition probability per unit time.

This integrates to N = N_0_ exp(−pT) where N_0_ = quantity at start time (T = 0).

Intracellular recordings have shown that thalamocortical neurons oscillate at different frequencies depending on the degree of their cell membrane polarization, which increases as sleep deepens and that there are corresponding firing rate modes in the thalamically projecting brainstem activating neurons [5], [11]. Applying this characteristic, the sleep-deepening phase can be described as a cascade of neuronal transitions beta→sigma→delta, with starting equations:
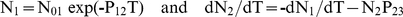
Solving for N_2_ and N_3_

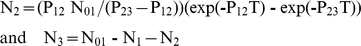
Where

N_01_ = initial number of neurons in beta mode = fixed generating population size and

N_1_, N_2_ & N_3_ = number of neurons oscillating in beta, sigma and delta mode respectively after time T

P_12_ = transition probability per unit time for neurons in beta mode→sigma mode

P_23_ = transition probability per unit time for neurons in sigma mode→delta mode

Since the exponential decline formula applies to each level of the cascading process, it follows that sigma maximum occurs while delta is still rising: initially N_2_ is zero and then increases since it is formed from the transition of type N_1_. But N_2_ neurons are themselves transforming to type N_3_. When the rate of formation of N_2_ neurons is equal to the rate of transformation, the N_2_ will pass through a maximum and then decline. At the same time, N_3_ can only continue to go up, though more slowly, still being supplied from the type N_2_ transformation.

For the reverse sleep lightening phase – moving *away* from deep sleep - with transitions delta→sigma→beta, similar formulae apply with transition probabilities P_32_ and P_21_. The initial values N_1_, N_2_ and N_3_ for any phase are equal to the end values of the preceding phase.

## Supporting Information

Figure S1 **Brainstem firing rate and EEG activity.** (a) Firing rate in a midbrain reticular neuron of the cat during repeated EEG desynchronisation-synchronisation transitions. The appearance of each spindle is paralleled by a marked decrease in firing rate. (b) Results from 12 such transitions confirming the relative instantaneity of the individual neuron time to transition from fast to spindle mode which on average is ∼1.5 seconds as compared to several minutes for the NREM episode duration (adapted from Steriade et al. [28] with kind permission of the publisher).
(TIF)

Figure S2 **Individual VLPO neurons change their firing rate mode within a few seconds.** Example of a VLPO neuron recorded during a sequence of sustained wake (a), wake to NREM transition (b), early NREM sleep (c), late NREM sleep (d) and back to wake (adapted from Szymusiak et al. [31] with kind permission of the publisher). The unit measurement data show the increase in firing rate as the neuron goes from wake (blue)→light sleep (green)→deep sleep (red). We see that transition from one firing mode to another (indicated by an arrow) takes place within a few seconds.
(TIF)

Table S1 **Goodness of fit of the NTP model to the delta sigma and beta time-courses measured by R^2^ (%).**
(DOC)

Video S1 **A Monte Carlo simulation of neuron transitions in the brainstem generating population during one NREM** ***towards*** **and** ***away*** **cycle – according to the NTP model.**
(MOV)

Text S1 **Findings at the neuronal level provide elements necessary for the establishment of tenable hypotheses relating the temporal progression of activity at the EEG level to that at sub-cortical level.**
(DOC)
