# Supplementary figures and images for: The Neuronal Transition Probability (NTP) Model for the Dynamic Progression of Non-REM Sleep EEG: The Role of the Suprachiasmatic Nucleus
Source: PLoS One. 2011 Aug 19;6(8):e23593. doi: 10.1371/journal.pone.0023593 (PMC3158790; doi:10.1371/journal.pone.0023593)

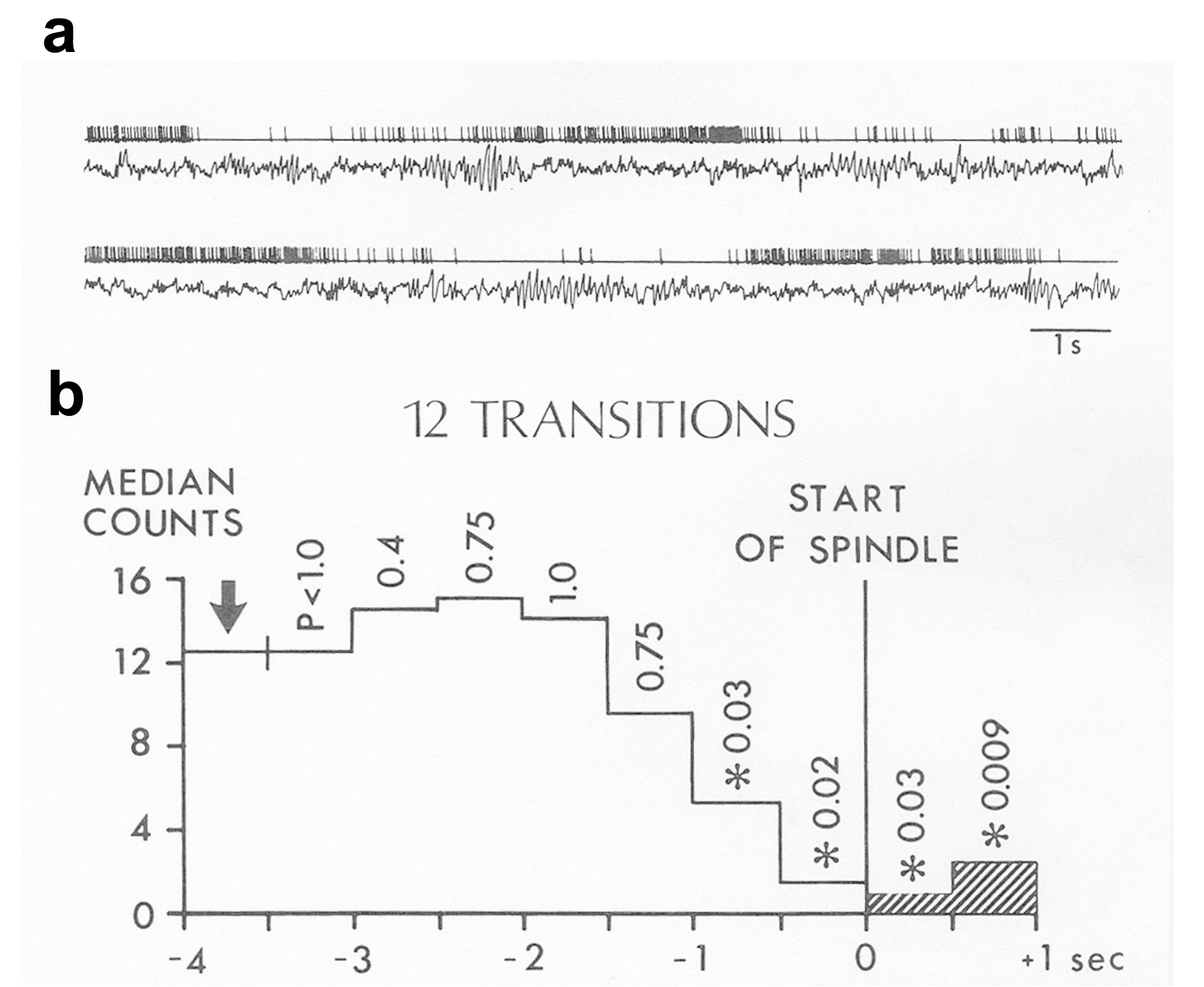

Supplement: Figure S1 — Brainstem firing rate and EEG activity. (a) Firing rate in a midbrain reticular neuron of the cat during repeated EEG desynchronisation-synchronisation transitions. The appearance of each spindle is paralleled by a marked decrease in firing rate. (b) Results from 12 such transitions confirming the relative instantaneity of the individual neuron time to transition from fast to spindle mode which on average is ∼1.5 seconds as compared to several minutes for the NREM episode duration (adapted from Steriade et al. [28] with kind permission of the publisher). (TIF) [file pone.0023593.s001.tif]

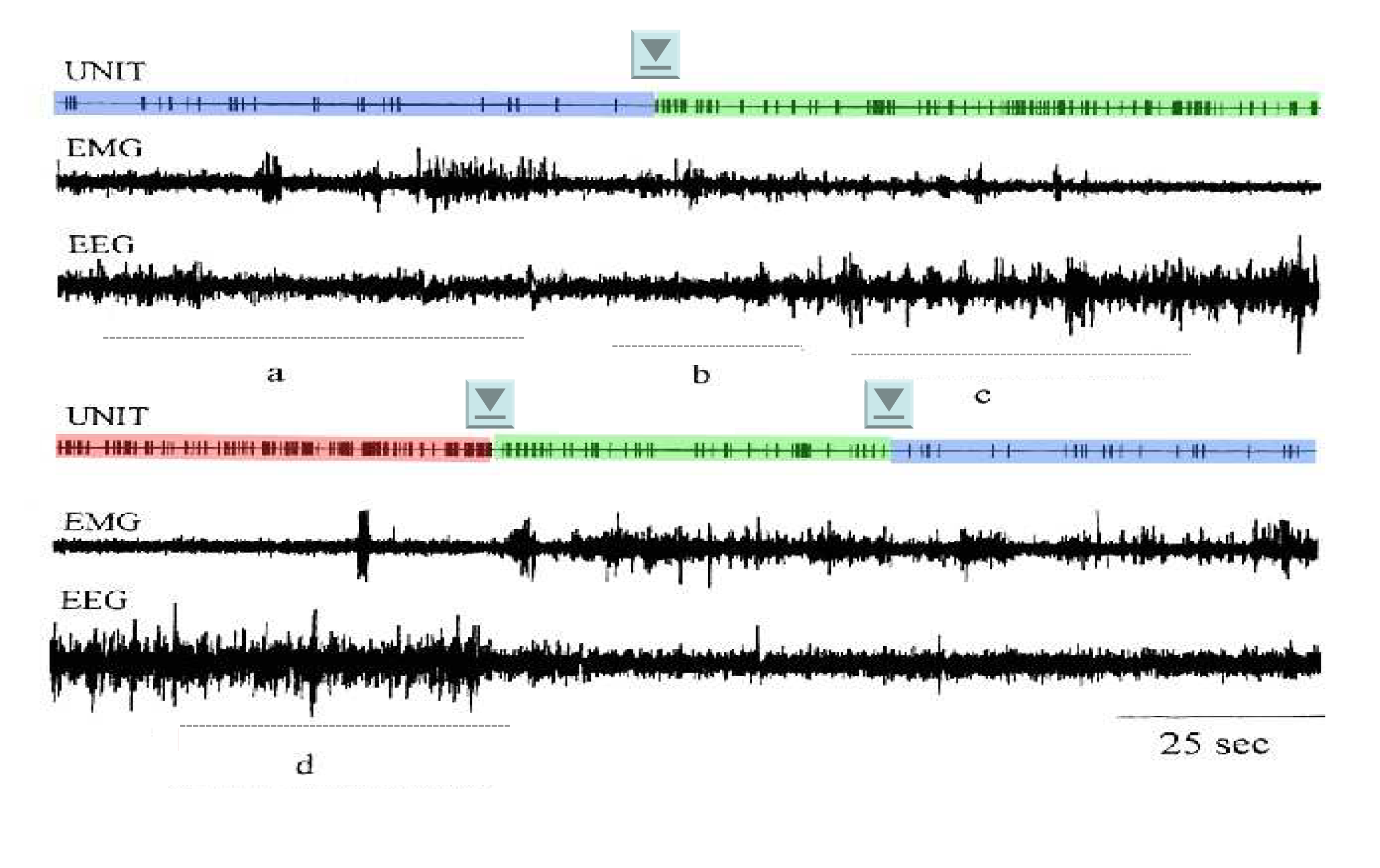

Supplement: Figure S2 — Individual VLPO neurons change their firing rate mode within a few seconds. Example of a VLPO neuron recorded during a sequence of sustained wake (a), wake to NREM transition (b), early NREM sleep (c), late NREM sleep (d) and back to wake (adapted from Szymusiak et al. [31] with kind permission of the publisher). The unit measurement data show the increase in firing rate as the neuron goes from wake (blue)→light sleep (green)→deep sleep (red). We see that transition from one firing mode to another (indicated by an arrow) takes place within a few seconds. (TIF) [file pone.0023593.s002.tif]
