# Supplementary material for: The Neuronal Transition Probability (NTP) Model for the Dynamic Progression of Non-REM Sleep EEG: The Role of the Suprachiasmatic Nucleus
Source: PLoS One. 2011 Aug 19;6(8):e23593. doi: 10.1371/journal.pone.0023593 (PMC3158790; doi:10.1371/journal.pone.0023593)
Supplement: Table S1 — Goodness of fit of the NTP model to the delta sigma and beta time-courses measured by R2 (%). (DOC) [file pone.0023593.s003.doc]

| **Table S1**. Goodness of fit of the NTP model to the delta sigma and beta time-courses measured by R^2^ (%). | | | | | | | | | | | | | |
| --- | --- | --- | --- | --- | --- | --- | --- | --- | --- | --- | --- | --- | --- |
|  |  |  |  |  |  |  |  |  |  |  |  |  |  |
|  |  | **NREM 1** |  |  | **NREM 2** |  |  | **NREM 3** |  |  | **NREM 4** |  |  |
| **Subject** | **delta** | **sigma** | **beta** | **delta** | **sigma** | **beta** | **delta** | **sigma** | **beta** | **delta** | **sigma** | **beta** |  |
| 1 | 85.1 | 32.5 | 67 | 89 | 77.1 | 40.1 | 46 | 20.1 | 47.5 | 64.7 | 34.2 | 59.9 |  |
| 2 | 78.8 | 40.9 | 60.2 | 62 | 22 | 20.2 | 67.3 | 26.4 | 20.9 | 45.3 | 42.2 | 25.7 |  |
| 3 | 80.3 | 50.2 | 55.9 | 73.2 | 19.6 | 22 | 67.1 | 41 | 23.3 | 68.9 | 37.3 | 29 |  |
| 4 | 87.4 | 78.6 | 87.1 | 64.9 | 17.5 | 16.4 | 85.2 | 35.2 | 46.7 | 62.7 | 57.2 | 40 |  |
| 5 | 88.6 | 63.6 | 84.7 | 85.7 | 28.6 | 71.1 | 89 | 26.2 | 56.9 | 53.9 | 27.2 | 21.6 |  |
| 6 | 85.1 | 53.8 | 70.9 | 91 | 58.9 | 64.6 | 54.5 | 31.7 | 13.7 | 84.2 | 45.2 | 31.9 |  |
| 7 | 80.6 | 42.8 | 66.6 | 69.2 | 23.5 | 34 | 90.8 | 52.6 | 70.3 | 28.3 | 60.1 | 22.8 |  |
| 8 | 88.5 | 48.9 | 49 | 94.6 | 29.2 | 16.2 | 54.1 | 17.3 | 21.1 | 70.3 | 30.5 | 23 |  |
| 9 | 80 | 46.8 | 62.3 | 86.1 | 55.9 | 34.3 | 78.2 | 56.4 | 45.7 | 60.4 | 42 | 18 |  |
| 10 | 88.8 | 43.4 | 80.9 | 72.5 | 22.6 | 42.5 | 84.3 | 44.6 | 56.4 | 11.6 | 23.8 | 22.4 |  |
| 11 | 77.2 | 39.4 | 67.6 | 89.2 | 48.6 | 76.2 | 80.1 | 23.3 | 33.3 | 66.1 | 30 | 48 |  |
| 12 | 88.2 | 41.1 | 34.9 | 70.5 | 28.5 | 23.3 | 61.6 | 18.3 | 17.3 | 63.1 | 39.2 | 20.6 |  |
| 13 | 85.1 | 27.1 | 40.1 | 68 | 51.1 | 47.9 | 75.1 | 39.9 | 37 | 60.7 | 37.2 | 56.3 |  |
| 14 | 78 | 28.4 | 60.4 | 74.4 | 58.3 | 64.5 | 54.7 | 21.5 | 41.5 | 45.5 | 27.5 | 33.1 |  |
| 15 | 79 | 46.1 | 38.8 | 80.4 | 45.8 | 37.4 | 93 | 41.6 | 54.4 | 63.1 | 30.6 | 29.1 |  |
| 16 | 88.6 | 69.4 | 68.7 | 74.6 | 34.3 | 59.1 | 71.2 | 36.6 | 39 | 86.2 | 46.1 | 42.4 |  |
| 17 | 92 | 64.5 | 51.1 | 79.5 | 56 | 40.2 | 54.4 | 50.2 | 24.1 | 70.2 | 47.5 | 48.1 |  |
| 18 | 87.3 | 43.7 | 76 | 85.3 | 23.1 | 28.1 | 62.9 | 27.1 | 21.7 | 81 | 37.9 | 54.8 |  |
| 19 | 80.4 | 68 | 60.3 | 82.5 | 26.3 | 42.1 | 75.5 | 36.3 | 47.3 | 52.4 | 20.4 | 14 |  |
| 20 | 88.1 | 51.5 | 91.1 | 53 | 28.2 | 55 | 84.1 | 29.2 | 38.1 | 30.5 | 19.6 | 30.4 |  |
| 21 | 90.3 | 38.5 | 36.6 | 86.1 | 48.6 | 52.2 | 50.2 | 13.4 | 17.3 | 80 | 27.5 | 40.2 |  |
| 22 | 85.5 | 37.4 | 81.4 | 71.3 | 41.5 | 22.3 | 78.3 | 49.1 | 37 | 60.4 | 32.9 | 16.2 |  |
| 23 | 85.2 | 25.2 | 71.9 | 90.4 | 42 | 33.8 | 79.1 | 31 | 21.5 | 47.7 | 54.9 | 14.4 |  |
| 24 | 88.6 | 37.1 | 70.2 | 62.5 | 31.3 | 40.4 | 86.3 | 24.1 | 67.7 | 28.2 | 31 | 27.5 |  |
| 25 | 84 | 53.4 | 80.2 | 78.2 | 23.7 | 32.6 | 84.6 | 28.4 | 56.7 | 39.1 | 39.8 | 41.6 |  |
| 26 | 90.5 | 42.2 | 63.1 | 82.1 | 26.6 | 43.3 | 82.9 | 24 | 56 | 62.5 | 15.7 | 20.6 |  |
| 27 | 89.5 | 54.5 | 91.3 | 85.1 | 25.1 | 52.2 | 83.2 | 26.7 | 35.7 | 42.7 | 24.6 | 40.6 |  |
| 28 | 83.3 | 36.2 | 43.5 | 76.3 | 29.7 | 29 | 70.7 | 40.5 | 24.8 | 69.1 | 38.2 | 19.2 |  |
| 29 | 80.2 | 43.6 | 70 | 88.3 | 28.2 | 61.9 | 77.2 | 38.5 | 72.5 | 58.5 | 38 | 44.4 |  |
| 30 | 83.5 | 60.9 | 35.1 | 80.3 | 33.8 | 36.1 | 77.3 | 22.7 | 21.1 | 51.7 | 25.6 | 25 |  |
|  |  |  |  |  |  |  |  |  |  |  |  |  |  |
| **Mean** | **85.5** | **47.9** | **67.2** | **80.2** | **36.7** | **42.2** | **75.9** | **32.5** | **39.6** | **59.2** | **35.6** | **32.0** |  |
|  |  |  |  |  |  |  |  |  |  |  |  |  |  |
| Mean values calculated using the Fisher z-transformed values | | | | | |  |  |  |  |  |  |  |  |
