# Supplementary material for: The Neuronal Transition Probability (NTP) Model for the Dynamic Progression of Non-REM Sleep EEG: The Role of the Suprachiasmatic Nucleus
Source: PLoS One. 2011 Aug 19;6(8):e23593. doi: 10.1371/journal.pone.0023593 (PMC3158790; doi:10.1371/journal.pone.0023593)
Supplement: Text S1 — Findings at the neuronal level provide elements necessary for the establishment of tenable hypotheses relating the temporal progression of activity at the EEG level to that at sub-cortical level. (DOC) [file pone.0023593.s005.doc]

**Findings at the neuronal level provide elements necessary for the establishment of tenable hypotheses relating the temporal progression of activity at the EEG level to that at sub-cortical level.**

Cellular neurophysiology provides evidence supporting the hypothesis formulated as early as 1930 [40, 41] that the hypothalamus controls the sleep and wake states. A population of sleep promoting neurons localised in the ventrolateral preoptic (VLPO) area of the hypothalamus has been identified [31, 42]. These GABAergic neurons are shown to be inhibited by wake-promoting monoaminergic and cholinergic neurotransmitters and it has been postulated that there exists a reciprocal inhibitory interaction between these neurons and wake-promoting neurons to which they project, localised in multiple arousal centres in the upper brainstem and diencephalon [43]. Thus under the influence of circadian factors, input from the retina [44] and the suprachiasmatic nucleus [45,46] together with the influence of homeostatic drive [47], the sleep-promoting neurons in the VLPO would increase their firing rate and in so doing inhibit the wake-promoting neurons, thereupon allowing for their own disinhibition and reinforced firing. Conversely, increased firing in the wake promoting neurons, inhibit VLPO neurons. This mutually inhibitory interaction between the sleep and wake promoting neurons is analogous to a ‘flip-flop’ (on-off) electrical switch circuit and is at the root of a sleep-wake switch model [15]. The initial increase in activity of the VLPO sleep-promoting neurons initiated by the wake-sleep switch continues to increase proportionally with sleep depth [31]. This progressive increase is mirrored by a progressive decrease in the firing rates of the wake-promoting neurons in the various arousal centres including those in the brainstem-thalamic activating system. As a result of the diminished excitatory input to the thalamus, thalamocortical neurons gradually become more hyperpolarized leading first to light sleep (spindle oscillatory mode) then at a more hyperpolarized level to deep slow wave sleep (clock-like delta mode). Increased excitatory input, on the other hand, depolarises the thalamocortical cells producing a transition to wake or REM sleep [5]. Thus there exists a direct parallelism between brainstem firing-rate change and sleep state progression. Moreover, decreased activity in the ascending activating systems leads to a progressive deafferentation of the forebrain, initiating at sleep onset the cortically generated slow (< 1Hz) oscillation, which reaches full development as sleep deepens and which is obliterated by brain activated states [48]. The depolarising component of this slow oscillation has a major role in triggering, shaping and synchronising thalamically generated rhythms (spindles and the clock-like component of delta waves) as well as fast oscillations, forming at the cortex the complex wave sequences observed on the EEG [49-51]. Thus under the modulatory control of the brainstem activating system a permanent dialogue is established between cortex and thalamus that allows the orderly appearance over the entire cortex of the various rhythms characterising NREM sleep progression. The cellular basis underlying the generation of the various EEG rhythms (spindles, delta waves and the slow oscillation) characterizing the NREM sleep stages has also been intensely studied. The sites of origin and the basic mechanisms that underlie each of these rhythms have largely been elucidated [9,11, 52-55]. These basic findings established essentially on the basis of state-dependent EEG characteristics and on events of the order of milliseconds to several seconds in duration, shed light on the general pattern of state progression operating across the NREM episode and provide the necessary elements to try and establish a link with the corresponding progression of activity at the EEG level.

(Reference numbers correspond to those in the main text)
